# Health care utilization related to the introduction of designated GPs at care homes in Denmark: a register-based study

**DOI:** 10.1080/02813432.2022.2057031

**Published:** 2022-04-01

**Authors:** Line Due Christensen, Claus Høstrup Vestergaard, Morten Bondo Christensen, Linda Huibers

**Affiliations:** a Research Unit for General Practice, Aarhus, Denmark; b Department of Public Health, Aarhus University, Aarhus, Denmark

**Keywords:** Home for the aged, caregivers, general practitioners, health services for the aged, aged, registries, Denmark

## Abstract

**Objective:**

To investigate the correlation between having designated general practitioners (GPs) in residential care homes and the residents’ number of contacts with primary care, number of hospital admissions and mortality.

**Design:**

A retrospective register-based longitudinal study.

**Setting:**

Forty-two care homes in Aarhus Municipality, Denmark.

**Subjects:**

A total of 2376 care home residents in the period from 1 September 2016 to 31 December 2018.

**Main outcome measures:**

We used two models to calculate the incidence risk ratio (IRR) for primary care contacts, hospital admission or dying. Model 1 compared the residents’ risk time before with their risk time after implementation of the designated GP model. Model 2 included only risk time after implementation and was based on calculations of successful (rate ≥60%) implementation.

**Results:**

Weighted by time at risk, the proportion of females across the two models ranged from 64% to 68%. The largest group was aged ‘85-94’ years. In Model 1, the mere implementation of the model did not correlate with changes in primary care contacts, hospital admissions, or mortality. Contrarily, in Model 2, residents living in care homes with successful implementation had fewer email contacts (IRR = 0.81, 95%CI: 0.68;0.96), fewer telephone contacts (IRR = 0.78, 95%CI: 0.68;0.90) and fewer hospital admissions (IRR = 0.85, 95%CI: 0.73;0.99), but more home visits (IRR = 1.70, 95%CI: 1.29;2.25) than residents living in care homes with lower implementation rates.

**Conclusion:**

The designated GP model seems promising, as a high implementation degree of the model correlated with a reduced the number of acute admissions, short-term admissions and readmissions. Future studies should focus on gaining deeper insight into the mechanisms of the designated GP model to further optimize the model.

**Key points**
- A new care model was introduced in Denmark in 2017, designating dedicated GPs to residential care homes for the elderly.
- Successful implementation correlated with significantly fewer hospital admissions, specifically for acute admissions, but also with fewer short-term admissions and readmissions.
- The implementation of the model correlated significantly with fewer e-mail and telephone contacts and with more home visits.
- Future studies should gain more insight into the mechanisms of the designated GP model to further optimize the model.

## Introduction

The number of care home residents is expected to increase due to the aging population. Care home residents are often frail, suffering from physical, cognitive and sensory impairments [1,2] and many have dementia [2]. Multimorbidity is common [3,4], increasing medication prescription rates are seen [5,6], and the average remaining life expectancy is limited [7]. These residents have a high need for health care services, including high rates of hospitalization, and increased risk of mortality [4,7,8]. Hospital admissions carry the risk of unplanned iatrogenic harm, reduced in functional abilities [9], hospital-acquired pneumonia [10] and worsening of dementia symptoms [11]. Thus, limiting unnecessary hospital admissions is beneficial for the care home residents. A Danish study on acute short-term hospital admission for elderly found that approximately 45% of the admissions of care home residents could have been replaced by other types of care [12].

The organization of medical care in care homes varies between countries. In some countries, the general practitioner (GP) takes care of the residents. This means that a high number of GPs are affiliated with each care home, and this requires the GPs to collaborate with several care homes and their staff [13,14]. This model has several negative effects, such as many hospitalizations, fragmented care and poor communication between patients, relatives, GPs and other health care professionals [15,16]. A positive aspect of continuing with the regular GP is that the established long-term relationship between the regular GP and the care home resident is preserved, despite the transfer to the care home setting. Improving the collaboration between GPs and care homes may reduce preventable admissions [14]. Some countries use various models to reduce the number of GPs at each care home. In Norway and in some parts of Germany, a designated GP provides primary care for all residents in each care home, whereas care home specialists are used in the Netherlands [14,17]. In Denmark, the implementation of the designated GP model started a few years ago [18]. So far, not all care homes have a designated GP affiliated [19]. To improve the already implemented designated GP model, more insight is needed into the impact of the model on the health-related outcomes in care homes residents.

We aimed to study the correlation between introducing designated GP in care homes and the residents’ number of contacts with general practice (i.e. daytime and outside office hours), hospital admissions (including readmissions, short and long-term admissions) and mortality.

## Methods

### Design and study cohort

**Figure 1.**
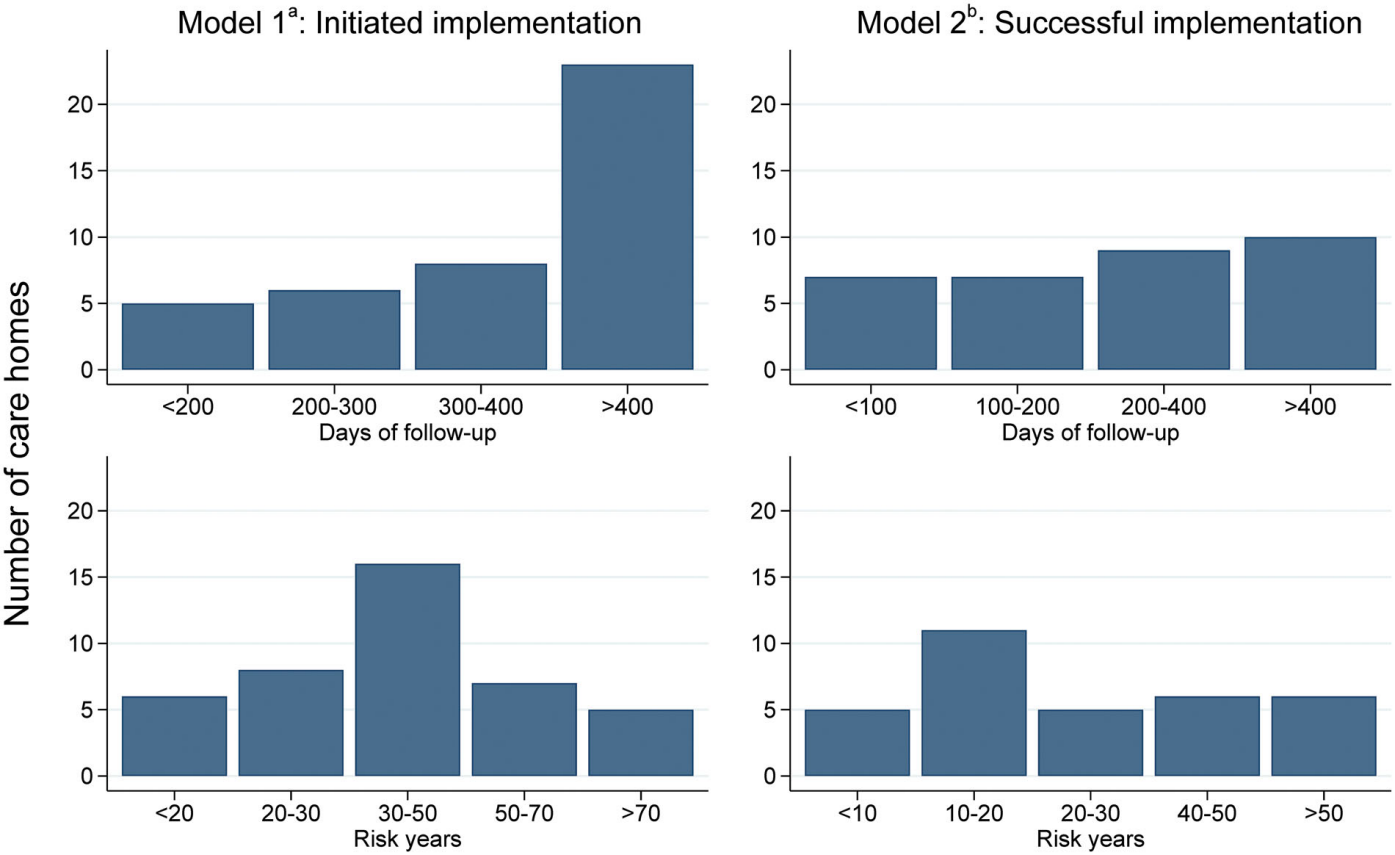
Days of follow-up and number of risk years. ^a^In Model 1, we compared the residents’ risk time before implementation with their risk time after implementation of the designated GP model. ^b^In Model 2, we compared the residents’ risk time before successful implementation (≥60%) with their risk time after successful implementation. The model included only risk time after implementation.

We conducted a retrospective register-based longitudinal study based on data from Aarhus Municipality in Denmark. These data were enriched with admission, primary care contacts, socioeconomic and comorbidity data extracted from the national registers at Statistics Denmark. The cohort included all residents aged ≥ 65 years who lived in one of the care homes affiliated with a designated GP in Aarhus Municipality at some point during the study period from 1 September 2016 to 31 December 2018. The follow-up period varied by care home according to implementation dates (Figure 1).

### Setting

Danish health care is a public system, which is financed through taxes; it offers free-of-charge access to health care through general practice. Moreover, GPs operate as independent contractors, and clinics are owned by the GPs. More than 99% of the citizens are listed with a specific GP, including care home residents [20,21]. According to Danish law, frail persons are suitable for care home residency if they need all day care. However, the actual allocation of residency is made by the local municipalities [22]. In Danish care homes, a team of nurses, care assistants and/or therapists with 2–4 years of basic education support and facilitate the well-being of the residents. From September 2017, the designated GP model was introduced in Danish care homes [19]. One GP or several are assigned to serve designated GP(s) in a care home while still maintaining their private practice [19]. The residents may keep their GP when moving into a care home, unless the care home is located too far away from their usual GP, or they actively choose someone else. New residents are encouraged, but not required, to register with the designated GP at the care home.

Care home, municipality and GP must agree on an implementation date before a designated GP can be affiliated with a specific care home.

### Outcome measures

We defined three outcome measures to investigate the implications of introducing the model of designated GPs in care homes: the number of contacts with general practice (in daytime and outside office hours), the number of planned and acute hospital somatic and psychiatric admissions (including acute admissions, readmissions, short and long-term admissions), and the mortality of the care home residents. These were defined as follows:

- Primary care contacts: total number of contacts with GPs, i.e. email contacts, telephone contacts, in-clinic consultations, home visits and out-of-hours contacts.
- Any admissions: any hospital admission (planned or acute).
  - Acute admissions: admission recorded as ‘acute’ in the Danish National Patient Register.
  - Short-term hospital admission: hospital admission lasting ≤24 h.
  - Long-term hospital admission: hospital admission lasting >24 h.
  - Readmissions: admission lasting >24 h and occurring within 30 d of discharge from previous admission.
- Hospital bed-days: number of days between any admission and discharge, i.e. the number of days spent at the hospital during the study period.
- Mortality.

In addition, the socioeconomic, demographic and comorbidity characteristics of residents were collected.

### Data sources

We collected data from the municipal care registration system and from national registries. Aarhus Municipality provided personal identification numbers for all care home residents during the study period and their care homes of residency, and the practice provider number of each of the affiliated designated GPs. The personal identification numbers and the practice provider numbers were used to link residents to a GP and to national register data. Data on contacts with general practice (daytime and out-of-hours) were obtained from the Danish National Health Service Register [21]. The Danish National Patient Register provided data on hospital admissions and discharge, including contact diagnoses [23]. In addition, the Danish Register of Causes of Death provided information on the date of death [24], whereas the Danish Civil Registration System and the Danish Education Registers were used to collect socio-demographic background information on care home residents (i.e. age, sex, marital status, urbanity and educational level) [25,26]. The implementation date of the designated GP model was obtained for each care home by requesting the date from the care home managers and the municipality separately; this information was added to the cohort data.

We categorized background characteristics into age groups (65–74, 75–84, 85–94 and >95 years), marital status (married, single), urbanity (urban, suburban/rural) and education level (<10, 10–15, >15 years, unknown). We used the hospital diagnosis codes to calculate Charlson Comorbidity Index to estimate comorbidity [27]. In addition, the diagnosis ‘dementia’, defined by dementia-related ICD-10 codes, was obtained from the Danish National Patient Registry for the period 1990–2018.

### Analytical approach

When comparing the implementation date recorded by the municipality and the care home managers with register data on recorded changes of GP assignment i.e. the actual implementation date for each individual resident, we found significant disagreement. Specifically, the actual implementation date was often considerably delayed compared to the date recorded by the municipality/care home managers. This delay in implementation was sure to misclassify a significant portion of the data as ‘post implementation’ when in reality it should be labeled ‘prior to implementation’, and consequently drag estimates toward the null. Therefore, we defined two models to investigate the effect of the designated GP model.

In Model 1, we compared the residents’ risk time before implementation of the designated GP model with their risk time after implementation, regardless of whether the care homes actually managed to reassign their population to their designated GP. This model is referred to as ‘Initiated implementation’.

In Model 2, we compared the residents’ risk time before successful implementation (defined as >60% of residents assigned to the designated GP) with their risk time after successful implementation. In addition to fighting misclassification, this model introduced heterogeneity in implementation dates, and thus helped distinguish between calendar effects (periods of three months) and implementation effects, as the original implementation dates (reported by the municipality and care home managers) clustered closely around September 2017. This model is referred to as ‘Successful implementation’.

### Statistical analyses

We defined the implementation date for each care home by using the dates from the municipality database and from the care home managers. In case of inconsistency, the latest of the two dates was used as the implementation date of the designated GP model. Due to death or relocation, most residents were included in the cohort in only part of the study period. Therefore, the summary statistics on resident characteristics and care home characteristics were weighted by the amount of total time spent at risk (i.e. risk time) and tabulated for each model. Thus, if a resident died after six months at the care home, this resident would only contribute with 25% to results compared to a resident living there for 2 years.

Next, all outcome measures (contacts to general practice, hospital admissions, mortality) were counted and analyzed with using Poisson regression. The two models were adjusted for the same set of covariates and calendar time in quarters, while taking into account the amount of risk time contributed by each resident, and were further adjusted for clustering at resident level. As the care home sector is subject to constant change, we measured time in quarters (not in years) to better account for any increased frailty in the care home residents. Model 1 suffered from misclassification error, i.e. unexposed risk time was mislabelled as exposed risk time, which tended to bias the effect estimates toward the null. Therefore, we introduced Model 2, which considered only risk time after reported implementation. Furthermore, we defined care homes with >60% of residents assigned to the designated GP, as having a successful implementation. This cut-off was somewhat arbitrary and based on consensus among the authors. Cut-offs of 50% and 70% were used for the sensitivity analysis and led to very similar results. The results are presented as incidence risk ratios (IRRs) with a corresponding 95% confidence interval (CI).

### Compliance with ethical standards

The project was listed in the record of processing activities at the Research Unit for General Practice in Aarhus in accordance with the provisions of the General Data Protection Regulation (GDPR). According to Danish legislation, ethical approval and informed consent were not required as the study was based on register data.

## Results

### Population

In Table 1, we present the background characteristics of the included populations in the two models, weighted by risk time. The proportion of females across the two models ranged from 64% to 68%. In both models, the largest age group was 85–94 years ranging from 39% to 40%. We found no difference in the prevalence of comorbidities (i.e. number of CCI) between the two models. The proportion of residents with dementia ranged from 31% to 39%.

**Table 1.** Characteristics of the study population (% of total risk years).

	Model 1^a^: Initiated implementation		Model 2^b^: Successful implementation	
	Yes	No	Yes	No
Total risk years (*n*)	1758	2269	897	861
Sex				
Female	66.7	67.1	67.5	65.9
Male	33.3	32.9	32.5	34.1
Age groups (in years)				
65–74	15.7	15.4	13.4	18.1
75–84	31.4	31.8	33.2	29.6
85–94	43.5	43.5	43.3	43.7
>94	9.3	9.3	10.1	8.5
Year^c^				
2016	0.0	25.7	0.0	0.0
2017	16.0	63.8	11.8	20.4
2018	84.0	10.5	88.2	79.6
Urbanity				
Urban	80.6	79.0	70.2	91.5
Suburban/rural	19.4	21.0	29.8	8.5
Marital status				
Married	21.7	19.9	23.7	19.5
Single	78.3	80.1	76.3	80.5
Education (in years)				
<10	41.9	42.8	43.2	40.5
10–15	37.2	35.4	38.0	36.2
>15	15.9	14.1	13.8	18.0
Unknown	5.1	7.7	5.0	5.2
Comorbidity (CCI)^d^				
0	24.9	25.0	25.9	23.9
1	42.1	42.8	41.8	42.5
2	21.3	20.4	21.0	21.7
+3	11.7	11.8	11.3	12.0
Dementia				
No	62.8	61.7	62.9	62.7
Yes	37.2	38.3	37.1	37.3
Unique persons (*n*)	2376	2607	1593	1,484
Unique care homes (*n*)	42	42	33	29

^a^In Model 1, we compared the residents’ risk time before implementation with their risk time after implementation of the designated GP model.

^b^In Model 2, we compared the residents’ risk time before successful implementation (≥60%) with their risk time after successful implementation. The model included only risk time after implementation.

^c^Indicates which year each group was entered.

^d^Measured by the Charlson comorbidity index (CCI).

### Model 1: Initiated implementation

**Figure 2.**
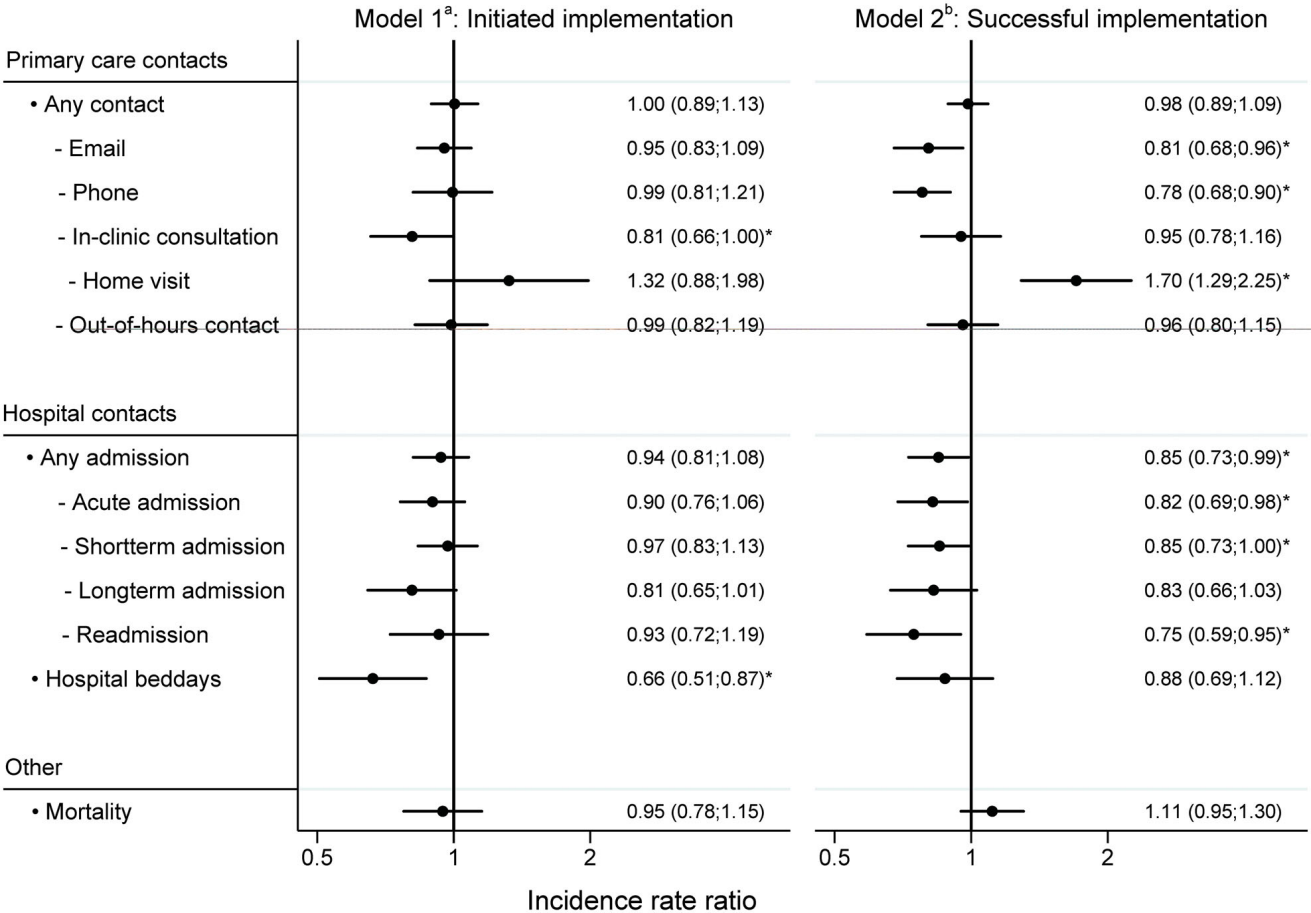
Forest plots of incidence ratios for primary care contacts, hospital contacts, and mortality for the two models (adjusted, with 95% confidence intervals). ^a^In Model 1, we compared the residents’ risk time before implementation with their risk time after implementation of the designated GP model. ^b^In Model 2, we compared the residents’ risk time before successful implementation (≥60%) with their risk time after successful implementation. The model included only risk time after implementation. *Significant results.

Adjusted analyses of the residents’ risk time before and after implementation of the designated GP model showed that residents living in care homes with a designated GP had fewer in-clinic consultations (adjusted IRR = 0.81, 95%CI: 0.66;1.00) and fewer hospital bed-days (adjusted IRR = 0.66, 95%CI: 0.51;0.87). On all other outcome the two groups did not differ significantly (Figure 2). Appendix 1 shows the raw numbers of contacts, hospitalizations and mortality.

### Model 2: Successful implementation

Adjusted analyses showed that residents living in care homes with successful implementation of the designated GP model had fewer e-mail contacts (adjusted IRR = 0.81, 95%CI: 0.68;0.96), fewer telephone contacts (adjusted IRR = 0.78, 95%CI: 0.68;0.90) and more home visits (adjusted IRR = 1.70, 95%CI: 1.29;2.25) from the GP compared to residents living in care homes with lower implementation rates (Figure 2). Moreover, fewer hospital admissions were found (adjusted IRR = 0.85, 95%CI: 0.73;0.99), for acute admissions (adj. IRR = 0.82, 95%CI: 0.69–0.98), short-term admissions (adj. IRR = 0.85, 95%CI: 0.73–1.00) and readmissions (adj. IRR = 0.75, 95%CI: 0.59–0.95). Mortality was also slightly higher in successfully implemented care homes, although statistically insignificant (Figure 2).

## Discussion

### Statement of principal findings

In this retrospective register-based longitudinal study, we investigated the correlation between introducing designated GPs at care homes and health care utilization and mortality of residents. Successful implementation of the designated GP model (≥60% of residents listed with the designated GP) was correlated with a reduction of 15% in overall hospitalizations, including acute contacts (18%), short-term admissions (15%) and readmissions (25%). Successful implementation of a designated GP did not affect the mortality of care home residents significantly.

### Strengths and limitations of the study

The main strength of this study is the use of high-quality data from the national registers at resident level supplemented by municipality data on care homes and designated GPs. This combination made it possible for us to identify residents of each care home and link residents to the designated GP. Registry data on the residents’ characteristics allowed us to correct for possible confounding due to differences between care homes, including the proportion of residents with dementia. However, dementia-related diagnoses could only be defined in the Danish National Patient Register, which provided data on hospital-based ICD-10 codes, and we do expect an underestimation of dementia cases in our population. A limitation in our study was that in Model 1, we compared the residents’ risk time before implementation with their risk time after implementation of the designated GP model. This approach revealed a few design flaws primarily difficulties with distinguishing between implementation effects and calendar effects, as the implementation dates clustered very closely around September 2017. We expected calendar effects to be rather large as the policies in this area are constantly changing, and the care homes accommodating an increasingly frail population. Another limitation was the seemingly low validity of the dates for implementation of the designated GP model. Yet, we were still able to follow the outcome measures over time, thereby being able to identify a possible effect of the model in a period after the implementation. Additionally, we included data from only one municipality, which resulted in limited power and may have affected generalizability. While residents in Danish care homes are a fairly homogenous group in terms of morbidity [7], the fact that this population stems from a mainly urban and suburban/urban area may complicate the generalizability to more rural regions of Denmark. Rural areas often have only a few GPs serving the population, including the local care homes. Thus, when a patient moves into a care home in a rural area, the patient is likely to already be affiliated with the care home GP, which could result in a smaller effect of moving into the care home. The Danish health care system is universal and offers expansive coverage. This may lower the generalizability to different settings, but urban areas in Denmark are still likely to be comparable to those of other Scandinavian and northern European countries, as argued by Achterberg et al. [28]. Furthermore, the data from the Danish National Patient Register were unavailable from 2019, due to a service update, which still delays the access considerably, and effectively limited the follow-up time of the study. Therefore, in addition the varying implementation dates of the care homes, the follow-up time after the start of the initiation of the implementation of the designated GP model was relatively short for a small number of included care homes (Figure 1).

### Findings in relation to other studies

In agreement with our findings, studies have found lower prevalence of hospitalizations in care homes with an in-house physician than in community-dwelling older people [29,30]. Further, a similar study from Denmark found that the designated GP model reduced preventable hospitalizations and readmissions [18]. Reilev et al. [7] argued that the decrease in hospitalizations is mostly assessed as a positive outcome, indicating that a substantial proportion of these hospital admissions could be preventable in a care home setting. There can be many reasons for the reduction in hospital admissions in care homes. Penders et al. [29] found that designated or in-house GPs tend to be more experienced and more confident in treating older residents and the GPs may be more cautious regarding hospital admissions, preferring palliative care within the care home setting for frail residents. In addition, designated GPs are likely to become more specialized, which could improve the residents’ health and optimize the medical treatment, thereby reducing hospitalizations [18]. Moreover, the lower hospitalization rate among care home residents with a designated GP could result from the GP’s professional assessment of how to avoid burdensome interventions, as hospital treatment is not always in the resident’s best interests [29]. In this study, it would seem to suggest that the number of hospital bed-days was slightly higher than the reduction in admissions, meaning that the average hospital stay was slightly longer among residents at care homes with full implementation. However, this difference was statistically insignificant. Poor communication between patients, relatives, GPs, hospitals and care home staff could be a reason for admitting a care home resident to a hospital [31]. In addition, regular home visits may positively influence the collaboration and communication in care homes to benefit both care homes and general practices [14]. Thus, the reduced number of telephone contacts and email correspondences may result from regular home visits. A disadvantage of the designated GP model could be that it often requires the long-term relationship between the care home resident and their regular GP to end, resulting in loss of important health care information and mutual understanding.

### Meaning of the study

Our study suggests that successful implementation of the designated GP model correlated with fewer acute contacts, short-term admissions and readmissions, but with similar number of hospital days. The designated GP model may have improved the collaboration between the care home staff and the designated GP, which could have positively affected continuity and quality of care [18]. Yet, the lower number of acute admissions, short-term admissions and readmissions could also be interpreted as inadequate quality of care, as the residents may not have been hospitalized timely enough. Further research could explore and develop the interprofessional collaboration between the designated GP and the care home staff to improve the quality of the model. In addition, future research should investigate causes for the lower number of hospitalizations and the unchanged number of hospital days. Furthermore, other models of care should be investigated, such as hospital-in-home services and municipal interdisciplinary care teams, including their suitability for older patients [32].

## Appendix 1

**Table A1.** Raw number for primary care contacts, hospital contacts and mortality for the two models.

	Model 1^a^: Initiated implementation		Model 2^b^: Successful implementation	
	Yes	No	Yes	No
Primary care contacts				
Any contact	77,984	60,717	30,387	30,330
Email	37,060	25,511	14,308	11,203
Phone	15,437	8226	4745	3,481
In-clinic consultation	7882	4624	2365	2,259
Home visit	8355	13,509	4587	8,922
Out-of-hours contact	7904	5716	2932	2,784
Hospital contacts				
Any admission	3770	2631	1432	1,199
Acute admission	1841	1258	694	564
Short-term admission	3058	2178	1182	996
Long-term admission	712	453	250	203
Readmission	1414	963	561	402
Hospital bed-days	4626	2640	1447	1,193
Other				
Mortality	833	607	278	329

^a^In Model 1, we compared the residents’ risk time before implementation with their risk time after implementation of the designated GP model.

^b^In Model 2, we compared the residents’ risk time before successful implementation (≥60%) with their risk time after successful implementation. The model included only risk time after implementation.
